# Prospective Evaluation of Safe Observation Period after Asymptomatic Penetrating Thoracic Injury: 1 Hour is Enough

**DOI:** 10.22114/ajem.v0i0.148

**Published:** 2019-06-22

**Authors:** Farhad Heydari, Babak Masoumi, Majid Zamani, Mohammad Nasr-Esfahani

**Affiliations:** Department of Emergency Medicine, School of Medicine, Isfahan University of Medical Sciences, Isfahan, Iran.

**Keywords:** Delayed Pneumothorax, Hemothorax, Penetrating, Pneumothorax, Thoracic Injury

## Abstract

**Introduction:**

The observation period was recently challenged by some studies; and it has been suggested that a 1-hour observation period may be sufficient to allow safe discharge in asymptomatic patients with penetrating thoracic injury (PTI) and normal initial Chest X-Ray (CXR).

**Objective:**

The current study was performed to investigate if in asymptomatic and hemodynamically stable patients with PTIs who has an initial normal evaluation, 1-hour observation interval is safe to detect clinically significant injuries and is it possible to discharge these patients safely after a negative Extended – Focused Assessment with Sonography in Trauma (E-FAST) at hour1 instead of hour 3.

**Method:**

This cross-sectional study was performed on asymptomatic patients with penetrating thoracic injury, referred to emergency department (ED) and normal initial CXR and the Extended Focused Assessment with Sonography in Trauma (E-FAST). The second E-FAST was done 1 hour after the first one and the third repeat E-FAST and control CXR then performed 3 hours post-injury. 24 hours follow up by phone call was done for each patient after discharge.

**Results:**

Finally, 117 patients with the average ages of 25.9 ± 7.8 years were enrolled of whom 92.5% were male. Eight patients developed PTX or HTX during first hour of observation that were diagnosed by E-FAST or CT scan requested by the in-charge physician. One hundred-nine patient completed E-FAST and radiograph studies at times zero, 1 h, and 3 h. One patient had a normal initial evaluation but demonstrated a PTX on the 3-h managed without intervention. The rate of delayed abnormality after an initially normal study was 7.7 % (9/117). No discharged patients returned to our ED with delayed manifestations of either PTX or HTX.

**Conclusion:**

The results of our study have shown that asymptomatic patients with PTI with negative initial evaluation and no deterioration at intervals, about 1 hour may be sufficient for detection of clinically significant pathology, considered for safe and early discharge.

## Introduction

Penetrating thoracic injuries (PTIs) account for 7% of all trauma admissions, and 16% of penetrating injuries (1). Approximately 15–30% of PTIs require surgery; so most of them are managed conservatively (2). The presentation of PTIs can vary widely, from stable patients with few complaints to hemodynamically unstable patients requiring immediate intervention, depending upon the damaged structures, the extent of the injury, simultaneous injuries and patient physical condition and mental state. A detailed physical examination may provide clues of life-threatening PTIs. Even apparently stable patients with PTIs can deteriorate precipitously and a rapid diagnosis and treatment must be performed to prevent life-threatening conditions (2, 3).

Asymptomatic patients with PTIs with an initial negative CXR presentation can be safely observed for probable development of a delayed PTX or HTX. A repeat examination and evaluation should be performed three hours after arrival based on current recommendations (4–6). If the reevaluation is negative, the patient can be discharged, with orders to return immediately if any concerning symptoms (e.g., increasing shortness of breath, painful swallowing, etc.) develop. The purpose of the second evaluation is to detect delayed PTX and HTX that assumed to be occurred in 3% of PTIs, but may affect as many as 14% (7).

Reducing the use of ED resource utilization in a busy ED is generally a reason to reduce the period of observation, as well as keeping asymptomatic patients in an ED for longer observation is a difficult task (4, 5, 8). Therefore, the observation period was recently challenged by some studies; and it has been suggested that in asymptomatic patients with PTIs and normal initial CXR, a 1-hour observation period may be sufficient to allow safe discharge (9, 10). The current study was performed to investigate that whether in asymptomatic and hemodynamically stable patients with PTIs who has an initial normal evaluation, 1-hour observation interval is safe to detect clinically significant injuries and is it possible to discharge these patients safely after a negative E-FAST at hour1 instead of hour 3.

## Methods

### Study design and setting

This cross-sectional study was performed from July 2017 to May 2018 on patients with PTI, referred to the emergency department (ED) of Alzahra and Kashani hospitals, two university educational hospitals, affiliated with Isfahan University of Medical Sciences in Iran. The study design was approved by the ethics committee of Isfahan University of Medical Sciences and the code IR.MUI.REC.1396.2.069 has been assigned. All patients were assessed and managed by the trauma team in accordance with standard protocols. Before doing the study, the patients signed the informed consent form.

### Study Population

Asymptomatic patients with PTI and a normal initial CXR were enrolled. Patients were excluded if they were hemodynamically unstable, had decreased lung sounds, presence of respiratory distress, obvious damage that just limited of the skin and subcutaneous tissue, intoxicated by alcohol or drugs with unreliable symptoms, left the hospital against medical advice before completing the observation period, or arrived at the ED after an hour of trauma event.

To calculate the sample size, taking 95% confidence interval (α=0.05), considering to 80% the prevalence of normal CXRs in patients with PTIs (15), and 1% maximum error (d=0.1), 96 people were estimated as a minimum required consecutive sample.

### Study protocol

All patients were managed by an emergency medicine resident based on ATLS guideline. After the primary survey, the patients underwent E-FAST at time zero by a trained emergency medicine specialist with bedside ultrasound machine (Philips Affiniti-50), using micro-convex transducer (2–4 MHz), immediately the first PA-CXR was performed in an upright position in deep inspiration. If no PTX or HTX was detected in this step, the patients were observed for 1 hour.

The second E-FAST was done 1 hour after the first one. The third E-FAST exam performed 3 hours after arrival. Finally, according to protocols for patients who their initial CXR was normal, the control CXR was requested three hours after the primary one. Further, chest computed tomography (CT) might be requested at the discretion of the in-charge physician.

All CXRs were evaluated by a radiologist who was blind to clinical and E-FAST findings. Data gathering was done by an emergency medicine resident. The clinical and demographic information (age, sex, and location of trauma) of the patients were recorded. All patients underwent serial clinical examination for at least three hours after the first CXR. Twenty-four hours follow up by phone call was done for each patient after discharge.

### Statistical analysis

The collected data were entered in SPSS Software (version 22.0). Based on CXR and CT scan (If done) findings, final diagnoses were determined. Chi-square test (for comparison between qualitative data) and Student t-test (for comparison between quantitative data) were used for data analysis. In all cases p<0.05 was considered as significant.

## Results

Of 137 eligible patients with isolated PTI, 120 patients were enrolled the study. Two patients left against medical advice before completion of all three E-FASTs. One patient with normal initial CXR had PTX in E-FAST at time zero, that confirmed with CT scan, requiring tube thoracostomy. Finally, 117 patients were asymptomatic and had no findings on initial CXR and E-FAST.

The mean age of the patients was 25.9 ± 7.8 (ranged from 13 to 56) years and included 111 men (92.5%) and 9 women (7.5%). Demographics and Clinical Characteristics of studied patients is reported in table 1. All PTIs were caused by stab wounds. Twenty-one patients sustained multiple penetrating injuries to the thorax. In total, the 120 patients had 143 thoracic wounds. Of all these, 44% were anterior thoracic wounds and 56% were posterior wounds (Table 1).

**Table 1:** Baseline characteristics of the patients (N = 120)

**Variable**	**Number (%)**
**Age (year)**	
≤ 16	9 (7.5%)
16 – 30	83 (69.1%)
31 – 45	23 (19.2%)
≥ 46	5 (4.2%)
**Sex**	
Male	111 (92.5%)
Female	9 (7.5%)
**Number of wounds**	
Single	99 (82.5%)
Two	19 (15.8%)
Three	2 (1.7%)
**Location of injury**	
Right anterior	38 (26.6%)
Right posterior	41 (28.7%)
Right axillary	5 (3.4%)
Left anterior	19 (13.3%)
Left posterior	31 (21.7%)
Left axillary	9 (6.3%)

Three patients found dyspnea in the first hour, that second E-FAST was positive in all of them. For two patients, CT scan was performed and they were undergoing tube thoracostomy at the discretion of in-charge physician, another patient was managed without tube thoracostomy.

**Figure 1:**
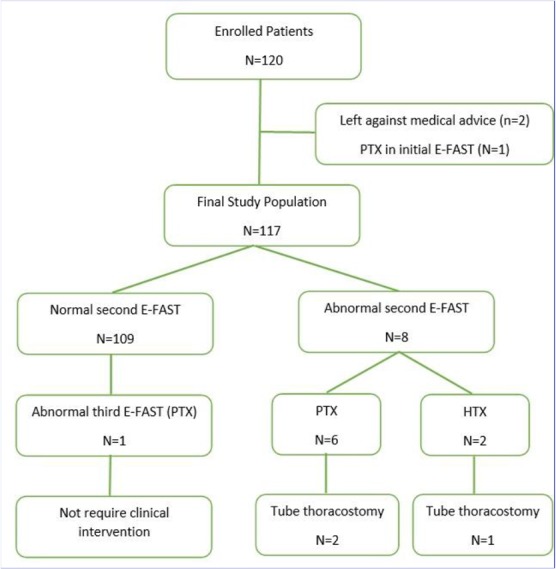
Study outline

Although, three patients developed PTX, and two patients developed HTX during the first hour. CT scan was performed in two patients and only one patient underwent tube thoracostomy and 4 patients managed conservatively, without any intervention and demonstrated no significant progression on further imaging. The remaining 109 patients had no positive finding on second E-FAST (Figure 1).

One hundred-nine patients completed E-FAST and radiograph studies at times zero, 1 h, and 3 h. One patient had a normal initial evaluation but demonstrated a PTX on the 3-h E-FAST and CXR. This single patient did not require any intervention. None of the remaining 108 patients developed a PTX or HTX on the 3-h study.

In this study, the rate of delayed abnormality after an initially normal E-FAST and CXR was 7.7 % (9 out of 117). PTXs accounted for the majority of observed abnormalities (7 out of 9, 77.8 %). The majority of the observed abnormality was diagnosed on the second E-FAST (8 out of 9, 88.9%) (Table 2).

**Table 2:** Findings on Serial E-FASTs

	**Initial**	**1 Hour**	**3 Hours**
**Pneumothorax**	1^*^	6	1
**Hemothorax**	0	2	0

* Initial CXR was normal

For each patient, a follow up phone call was performed 24 hours after discharge. Only 97 patients were available for follow-up, none of them reported problems. None returned to our ED with delayed manifestations of either PTX or HTX.

## Discussion

The current study shows that observing patients for 1-hour period with a secondary E-FAST on hour 1 is as sensitive as a 3-hour observation period with serial radiographs or E-FAST for the detection of delayed PTX or HTX require intervention in patients with asymptomatic PTIs.

In our study, second repeat E-FAST, at time 1 hour detected all clinically significant pathology in asymptomatic patients with normal initial E-FAST. The majority of the observed abnormality were diagnosed on the second E-FAST (8/9, 88.9%). A PTX developed in 1 of 109 (<1%) of the patients on third E-FAST at time 3 hours, but not require clinical intervention or demonstrated significant subsequent progression. Second repeat E-FAST also successfully detected all cases of delayed HTX (2/117, 1.7 %). No study patients returned to our ED with delayed manifestations of either PTX or HTX. So, a normal E-FAST after 1-hour observation period strongly supports the absence of significant underlying pathology and suggest that patients may be safely discharged from busy EDs.

Most authors agree on the necessity of observation and serial evaluation in asymptomatic PTIs patients, but there is disagreement about the length of time they are observed and type of evaluation. Older studies recommended that patients with penetrating thoracic injury, at the time of arrival and 3 hours later, take CXRs. More recent studies, decreased this period to 1 h in PTIs (4–6, 9, 10).

Berg et al. prospectively evaluated asymptomatic patients with PTI for 1 h follow up period. Eighty-seven patients were enrolled and completed initial, 1 hour, and 3-hour CXR. Four of 87 (4.6 %) demonstrated radiographic abnormalities on “early” repeat CXR at time 1 hour. Two patients had PTX, successfully managed without intervention; and two patients had HTX, subsequently undergoing tube thoracostomy. Two more patients (2.3 %) developed PTX between 1 hour and 3 hours on “delayed” CXR, both successfully observed without intervention. They concluded that in asymptomatic patients with penetrating thoracic injury and normal initial CXRs, “early” repeat CXR, at intervals approaching 1 h, appears sufficient to exclude clinically significant pathology and to allow safe patient discharge (10).

More recently, Seidzadeh Gooklan et al. prospectively evaluated 68 patients who had initial, 1 hour, and3 hour PA-CXR after PTIs. There was 100% concordance among CXRs performed at hours 1 and 3 in the study population. None of the patients showed clinical deterioration or PTX in CXR at hour 1 if remained asymptomatic during the first hour of observation (9).

In the present study, the rate of delayed abnormality after an initially normal E-FAST and CXR was 7.7 % (9/117). PTXs accounted for the majority of observed abnormalities (7/9, 77.8 %). Our study finding showed an overall 5.98 % (7/117) incidence of delayed PTX, an incidence within the range of 1.1–6 % reported by recent studies (4–6, 9, 10). Only one patient developed PTX between 1 hour and 3 hours on third E-FAST and no required clinical intervention.

Our findings were similar to other studies that detected delayed PTX by 1-hour observation. When all available data comparing 1-hour and 3-hour evaluations are pooled, a total of 264 patients with asymptomatic PTI have had initial, 1-hour, and 3-hour studies. There were nine (3.4%) delayed PTXs and HTXs between 0 hour and 1 hours whereas there were 3 between 1 hours and 3 hours in asymptomatic patients (Table 3). In contrast to Seidzadeh Gooklan et al. and similar to Berg et al., we found delayed PTX in the 3rd-hour study; fortunately, in these studies, no clinically significant PTXs were detected and those PTX cases found in the 3rd hour did not need any clinical intervention (9, 10).

**Table 3:** Literature comparing findings on 1 and 3 hours in asymptomatic patients

	**n**	**1 Hour**	**3 Hours**
**Gooklan et al.**	68	0	0
**Berg et al.**	87	4	2
**Present study**	109	5^*^	1
**Total**	264	9	3

* Also, there were 3 findings in symptomatic patients

### Limitation

One of the limitations of the present study was the first hour evaluation. In patient with delayed PTX, only the E-FAST was performed at the first hour, it was impossible to say with certainty that the patient had no PTX at this time. If CT scan was applied, it was probable that occult PTXs or HTXs was detected and it could be lead to change the findings. Also E-FAST dependency on operator proficiency.

## Conclusions

The results of our study have shown that asymptomatic patients with PTI with negative initial evaluation, and no deterioration at intervals approaching 1 hour may be sufficient for detection of clinically significant pathology, considered for safe and early discharge. Our study suggests that extending the time between initial and final evaluation to 3 hours in these patients provides no additional significant information that is not available on the 1hour evaluation and shortening the observation period after asymptomatic PTI to 1 hours could be safe, maybe cost-effective, and may help the quality of care benefits in busy EDs.
